# Effect of Electrolyzed Alkaline-Reduced Water on the Early Strength Development of Cement Mortar Using Blast Furnace Slag

**DOI:** 10.3390/ma13204620

**Published:** 2020-10-16

**Authors:** Taegyu Lee, Suna Kim, Sun-Gyu Park

**Affiliations:** 1 Department of Fire and Disaster Prevention, Semyung University, 65 Semyung-ro, Jecheon-si, Chungbuk 27136, Korea; ltg777@semyung.ac.kr; 2 Department of Architectural Engineering, Mokwon University, 88 Doanbukro, Seogu, Daejeon 35349, Korea; ksa8543@mokwon.ac.kr

**Keywords:** industrial by-product, blast furnace slag, electrolyzed alkaline aqueous solution, compressive strength, hydration reaction

## Abstract

This study evaluated the use of electrolyzed alkaline-reduced water instead of an alkaline activator for the production of a strong cement matrix with a large blast furnace slag replacement ratio. The flexural and compressive strength measurements, X-ray diffraction analysis, and scanning electron microscopy images of the cement matrices produced using electrolyzed alkaline-reduced water and regular tap water, and with blast furnace slag replacement ratios of 30 and 50% were compared to a normal cement matrix. The cement matrix produced using electrolyzed alkaline-reduced water and blast furnace slag exhibited an improved early age strength, where hydrate formation increased on the particle surface. The cement matrix produced using electrolyzed alkaline-reduced water exhibited a high strength development rate of over 90% of ordinary Portland cement (OPC) in BFS30. Therefore, the use of electrolyzed alkaline-reduced water in the place of an alkaline activator allowed for the formation of a very strong cement matrix in the early stages of aging when a large blast furnace slag replacement ratio was used.

## 1. Introduction

Cement is an important material that is extensively used in construction. The manufacture of cement currently accounts for approximately 18% of the total CO_2_ emissions of the manufacturing sector [1,2,3,4]. Consequently, the cement industry has been subject to numerous domestic and international sanctions in an effort to reduce its CO_2_ generation, which is a major cause of global warming. Therefore, prominent research in the domestic and foreign construction industries has focused on the reduction of CO_2_ [5,6,7].

Methods have been developed to substitute a portion of cement with industrial by-products, including blast furnace slag (BFS), fly ash, and silica fume, in the hydration reaction mechanism of cement [8,9,10]. As a concrete binder, mineral admixture is widely used in construction sites because it has many advantages such as increased workability of concrete, improved watertightness, and increased durability. It is also useful for the construction of sound structures through the reduction of the hydration heat and the cracking of concrete [11,12,13,14,15,16,17].

**Figure 1:**
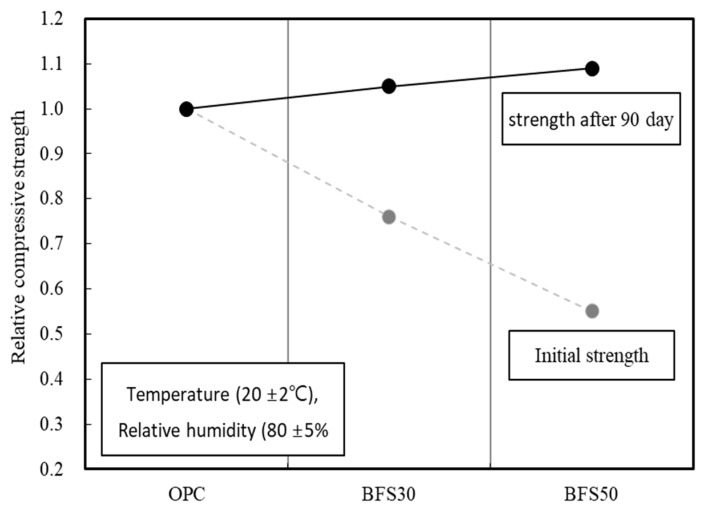
Initial and long-term strength development of mortar according to the BFS (BFS) replacement ratio [11,12,13,14,15].

Blast furnace slag has a high CaO content, which is required for the hydration reaction of cement, and has thus become very popular. BFS is produced by quenching and finely grinding the by-products obtained when melting iron ore, iron pellets, coke, or flux. Fast quenching of BFS leads to the formation of an amorphous layer on the particle [5,18]. This layer does not react immediately upon contact with water but readily dissolves in the strong alkaline Ca(OH)_2_ produced by the reaction between cement and water during hydration. Therefore, BFS is considered to be a latent hydraulic material, which strengthens via a reaction between CaO and binding water once the amorphous layer is removed [19,20]. Several studies investigated the strength development attributed to the reaction of the BFS organic surface film, where lower initial compressive strength has been reported when BFS is used as a cement substitution [21,22,23]. Specifically, the compressive strength of a cement matrix substituted with BFS linearly decreased at initial strength (Figure 1). During longer term aging of 28 days, the compressive strength increased compared to ordinary Portland cement (OPC), although the effect was small compared to the initial loss in strength [5,24]. Therefore, a cement matrix with BFS may severely affect construction productivity due to the delay in initial strength development. This may limit the usability of BFS in buildings that require rapid construction process management [25,26,27].

The initial strength of BFS can be improved using strong alkaline activators (e.g., NaOH or Na_2_SiO_3_) to rapidly dissolve the amorphous layer [28,29,30]. The use of an alkaline activator could allow for the use of larger amounts of BFS, as well as improve the initial strength of a matrix containing BFS. However, because these alkali activators are high-purity chemicals, production costs are high. Furthermore, high-purity strong alkaline activators may prevent complete bonding with the cement matrix, often causing elution of the alkali.

Binding water is an inexpensive material that can greatly impact the cement matrix. Chakraborty and Jo [31,32] studied the initial properties of concrete produced using hydrogen-enriched water, while Chikh [33,34] used electrolyzed water to improve the initial strength of a cement matrix. These studies demonstrated an initial improvement in strength of the cement matrix (compressive and flexural strength), but some limitations were reported for the use of large BFS substitutions with binding water.

The electrolyzed alkaline-reduced water used in this study is strongly alkaline electrolyzed water generated by purifying and then electrolyzing tap water with direct current. When strongly alkaline electrolyzed water is used as concrete binding water, additional costs, and high-concentration NaOH are not required; thus, the cost of hardened concrete can be considerably reduced. In addition, because a separate additional process is not required, productivity is improved and chemical reactions such as that of cement and BFS can be accelerated, thereby minimizing temporal and environmental concerns.

Research and development with respect to the improvement of the initial strength of cement matrices using binding water is still in the early stages, but exhibits potential as an innovative alternative to addressing various issues in construction.

This study used electrolyzed alkaline-reduced water to examine the improvement of the initial strength of the cement matrix mixed with BFS. Thus, we intend to review the use of electrolyzed alkaline-reduced water to secure early strength while reducing the use of OPC as much as possible.

## 2. Experimental Work

### 2.1. Materials

**Figure 2:**
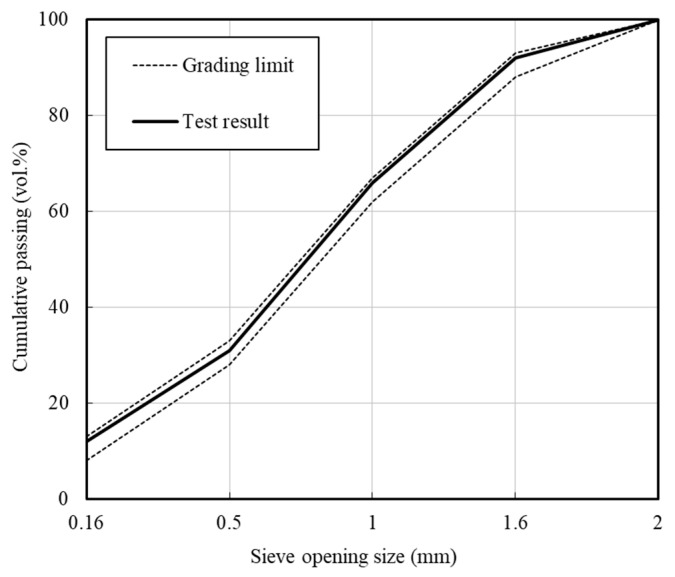
Gradation sieve analysis of ISO standard sand.

The chemical compositions of the OPC and BFS are given in Table 1. OPC mainly consisted of CaO, SiO_2_, and Al_2_O_3_, while BFS contained more SiO_2_, Al_2_O_3_, and MgO than OPC. The physical properties of the OPC, BFS and the fine aggregate are given in Table 2. Specifically, the OPC used in this study was Type-1 Portland cement (3150 kg/m^3^, fineness: 330 m^2^/kg), the BFS served as a mineral admixture (density: 2250 kg/m^3^, fineness: 360 m^2^/kg), and the fine aggregate was International Organization for Standardization (ISO) standard sand (density: 2620 kg/m^3^, SiO_2_: 99%, 0.08 mm passage amount: 0.04%) [35]. The sieve distribution curve of the fine aggregate is given in Figure 2.

**Figure 3:**
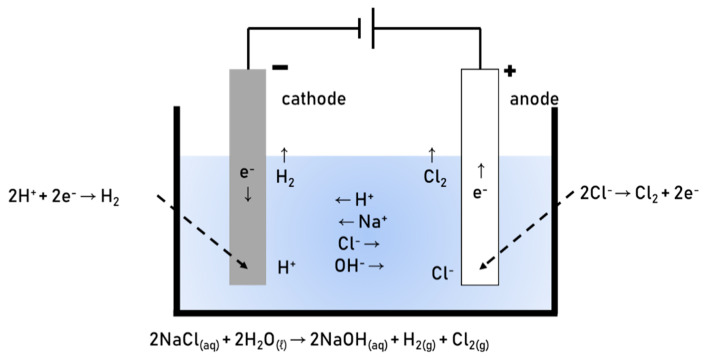
Electrolysis of alkaline-reduced water.

Tap water and electrolyzed alkaline-reduced water were used as binding water. The electrolyzed alkaline-reduced water served as a substitute for the alkaline activator and was obtained by electrolyzing pure water (Figure 3). Pure water has very low conductivity, thus an electrolyte (NaCl) was added. The water and dissolved electrolyte flowed to both electrodes, where the solution dissociated into Cl, H, Na, and OH ions. The former two were removed as gases, Cl_2_ and H_2_, while the Na and OH ions remained in the aqueous state, thereby producing an aqueous NaOH solution.

The pH of the NaOH aqueous solution produced using NaCl as an electrolyte was 12.5–13.0, which is very strongly alkaline, as measured using a pH meter (pH Tester 30, EUTECH, Seoul, South Korea). (The variation in pH is due to the fact that the solution was taken from an industrial process.) The electrolytic process proceeded according to the following chemical equations:

Oxidation reaction (Cathode): 2Cl^−^ → Cl_2_ + 2e^−^ (1)

Reduction reaction (Anode): 2H_2_O + 2e^−^ → H_2_ + 2OH^−^ (2)

2NaCl + 2H_2_O → 2Na^+^ + 2OH^−^ + H_2_ + Cl_2_ (3)

### 2.2. Experimental Design and Mix Proportions

The experimental factors and levels are given in Table 3. The water-to-total-binder (W/B) ratio of 0.50 was selected based on preliminary testing, where BFS was used as the binding material, and alkaline-reduced water and regular tap water were used as the binding water. The substitution ratio of BFS in the mortar was 30 and 50 wt%. The cement matrix underwent dry curing for 24 h at a humidity of 80 ± 5% and temperature of 20 ± 2 °C, followed by water curing until the flexural and compressive strength measurements conducted on either day 5 or 10. X-ray diffraction (XRD), and scanning electron microscopy (SEM) analyses were conducted to analyze the hydration properties of the cement matrix. The mixing proportions for the mortar are given in Table 4, where the binder-to-sand ratio was the standard mixing proportion of 1:3.

### 2.3. Test Methods

Table 5 shows testing of the mortar hardening properties. The flexural strength test was conducted according to the ASTM C348 standard test [36], while the compressive strength test was conducted according to the ASTM C109/C109M standard test [37], where 40 × 40 × 160 mm samples were used. The strength of the cement matrix was measured on days 5 and 10 after the manufacturing of the sample. SEM and XRD analyses were performed at the end of hydration.

Microanalysis of the mortar was conducted using SEM according to the ASTM C1723 standard test [38] and XRD according to the ASTM C1365 standard test [39]. SEM was performed using a Genesis-2020 microscope (Emcrafts, Gwangju-si, South Korea) at 20-kV voltage, and XRD was performed using an X′pert3 Powder PW 3050 instrument (Malvern Panalytical, Seongnam-si, Korea) at 60 kV and 50 mA.

## 3. Experimental Results and Discussions

### 3.1. Strength Properties

**Figure 4:**
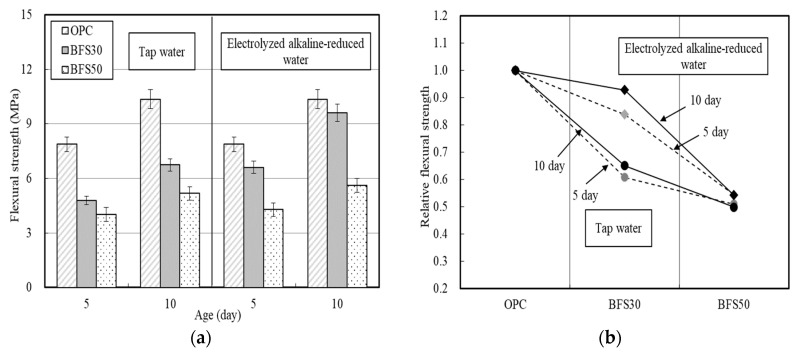
Flexural strength of cement matrices according to BFS replacement ratio—(**a**) flexural strength; (**b**) ratio of flexural strength relative to OPC.

The flexural strength of the mortar was evaluated according to the BFS replacement ratio (Figure 4). The flexural strength of the cement matrix was higher when produced using the electrolyzed alkaline-reduced water compared to tap water. The flexural strength of BFS30_EW was 84% of that of OPC on day 5, and improved to 93% by day 10. However, BFS50_EW exhibited flexural strength of only 60% of that of OPC, which was only slightly higher than BFS50_TW.

**Figure 5:**
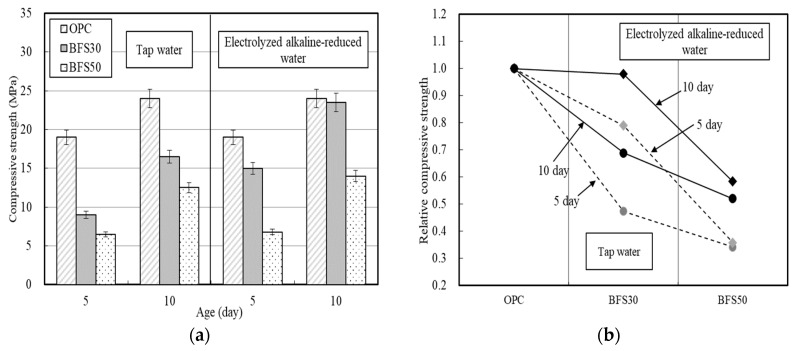
Compressive strength development of cement matrices according to BFS replacement—(**a**) compressive strength; (**b**) ratio of compressive strength relative to OPC.

The compressive strength was also evaluated according to the BFS replacement ratio (Figure 5). Similar to the flexural strength trend, the compressive strength of mortar prepared using electrolyzed alkaline-reduced water was higher than when tap water was used, where BFS30_EW was 79 and 97% of that of OPC on days 5 and 10, respectively.

However, unlike the flexural strength test results, BFS50_TW exhibited significantly reduced strength, which was only 34% of that of the OPC on day 5. Figure 4b and Figure 5b reveal that the relative strength of the cement matrix using electrolyzed alkaline-reduced water is higher than that using tap water. In particular, even if electrolyzed alkaline-reduced water is used, the increase in the strength of BFS50 is smaller than that of BFS30.

Berhan [40] reported that mortar with a BFS replacement ratio of 50% exhibited an improved compressive strength over the first 10 days with increasing molar concentration of NaOH, while the long-term compressive strength decreased. The compressive strength values reported in previous studies were similar to those reported in the current study when a 5 M NaOH alkaline activator was used, although the pH of this solution was higher than that of the electrolyzed alkaline-reduced water. Therefore, the use of electrolyzed alkaline-reduced water as binding water had a similar effect as that on addition of 5 M NaOH.

### 3.2. X-ray Diffaction Analysis

**Figure 6:**
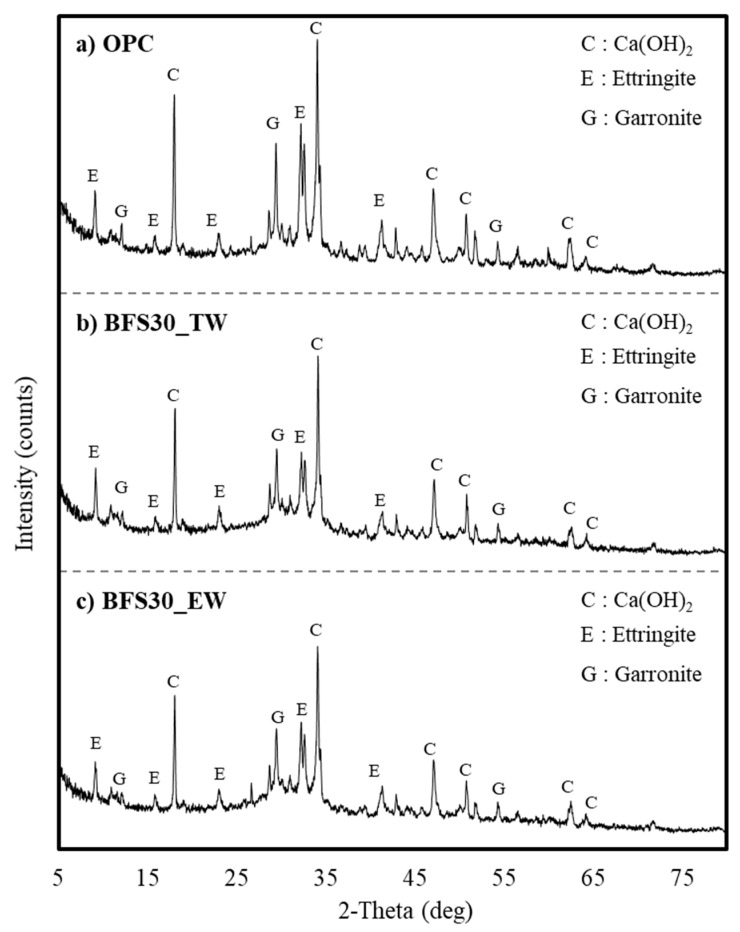
XRD analysis of cement matrices on day 5—(**a**) OPC (with tap water); (**b**) BFS30_TW and (**c**) BFS30_EW.

The XRD of the OPC and BFS30 cement matrices on day 5 exhibited 2-theta peaks at 7.2, 15.1, 22.9, 32.6, and 40.8°, which were attributed to ettringite from the raw material cement (Figure 6).

The peaks at 17.2, 33.1, 47.2, 51.2, and 63.3° were related to Ca(OH)_2_, while those at 8.7, 29.3, and 54.8° were attributed to garronite. BFS30 exhibited slightly lower value for the peaks related to Ca(OH)_2_ compared to OPC, due to the substitution of 30% of the cement with BFS. This is attributable to the small amount of CaO according to the slag replacement rate and the slow reaction of BFS.

**Figure 7:**
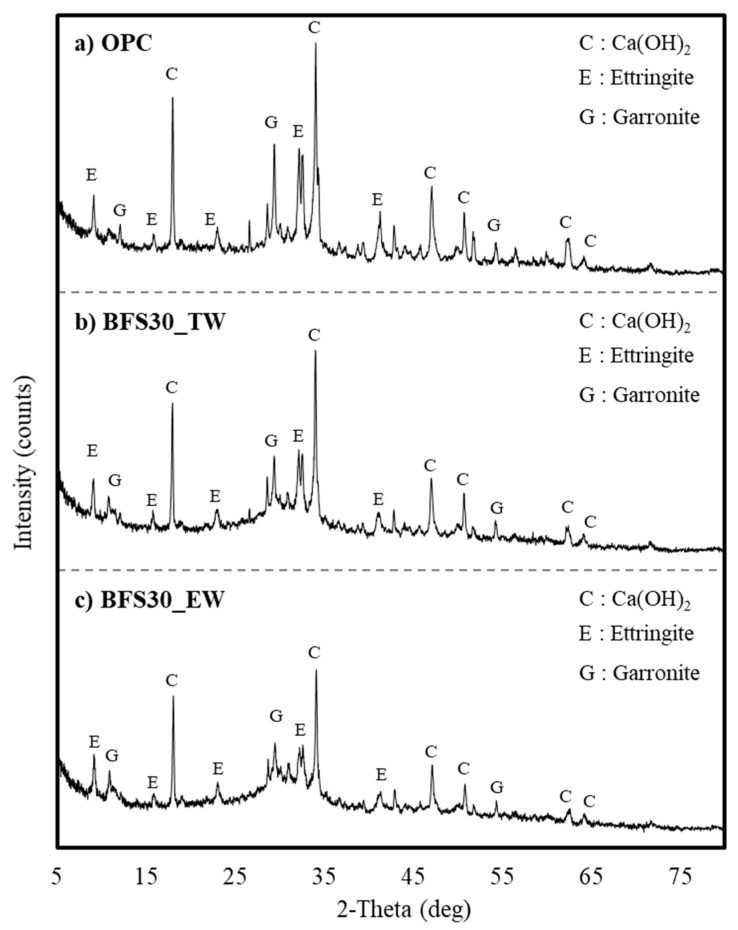
XRD analysis results of cement matrices on day 10—(**a**) OPC (with tap water); (**b**) BFS30_TW; and (**c**) BFS30_EW.

No significant differences in Ca(OH)_2_ content were exhibited in the XRD patterns of BFS30_TW and BFS30_EW on day 5. The peaks attributed to Ca(OH)_2_ generally increased in the OPC sample by day 10 (Figure 7). However, the XRD patterns of BFS30 did not exhibit changes between days 5 and 10.

In the case of OPC without BFS substitution, the peaks of Ca(OH)_2_, ettringite, and garronite had a high intensity, and BFS30_TW and BFS30_EW showed similar patterns. Therefore, the XRD analysis of concrete mixed with BFS confirmed that the difference according to the type of water was not large. Therefore, when electrolyzed alkaline-reduced water is used, it is difficult to clearly conclude that the increase in strength is due to the increase in basicity; thus, the reaction products must be additionally analyzed.

### 3.3. Scanning Electron Microscopy

**Figure 8:**
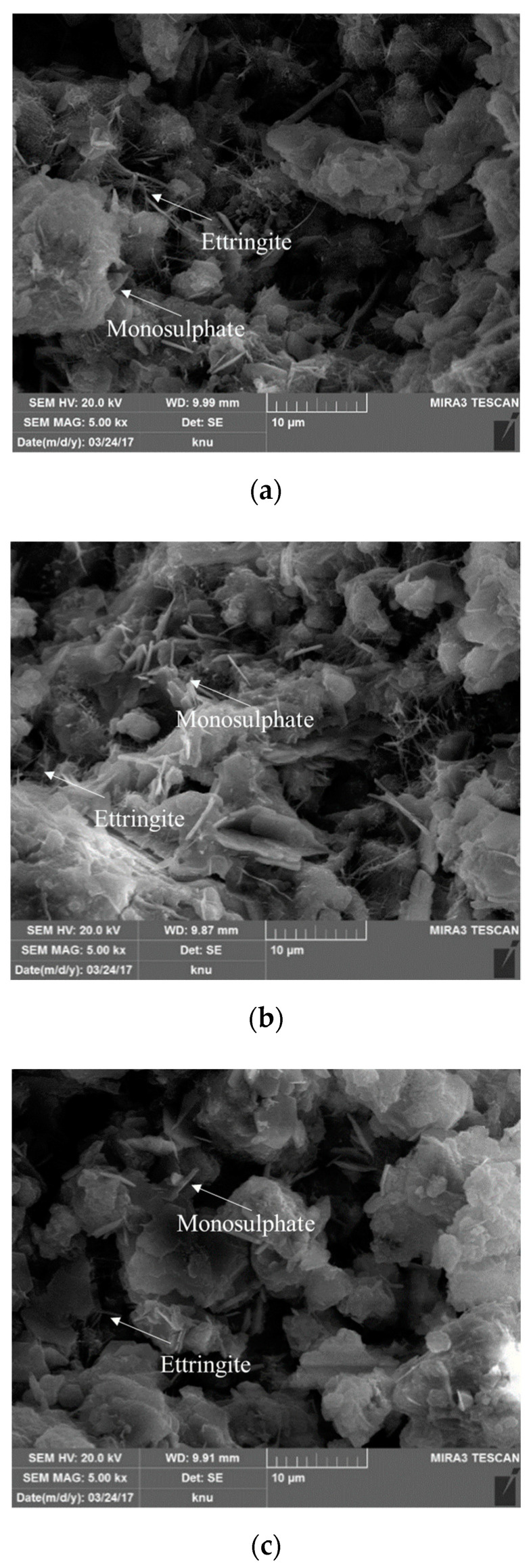
SEM analysis of cement matrices on day 5—(**a**) OPC (with tap water); (**b**) BFS30_TW; and (**c**) BFS30_EW.

**Figure 9:**
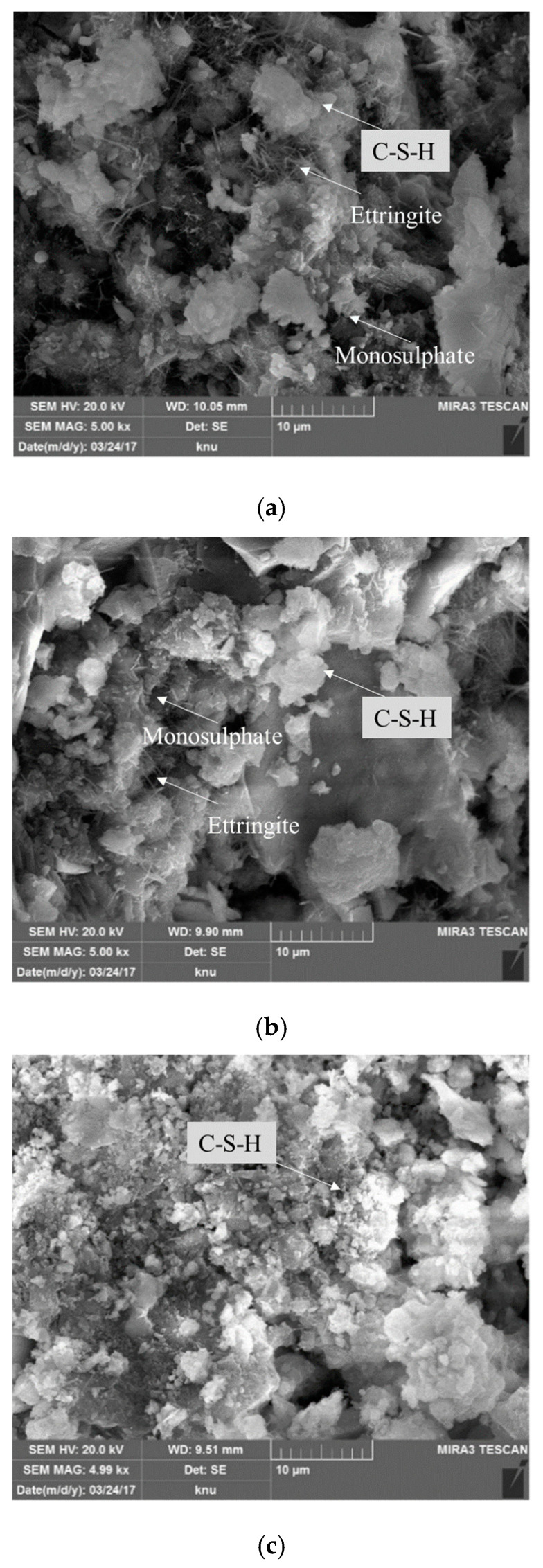
SEM analysis of cement matrices on day 10—(**a**) OPC (with tap water); (**b**) BFS30_TW; and (**c**) BFS30_EW.

The SEM images of the OPC and BFS30 cement matrices on days 5 and 10 are presented in Figure 8 and Figure 9. The OPC sample exhibited needle- and plate-shaped hydrates, which are commonly formed in typical cement matrix structures (Figure 8a).

BFS30_TW exhibited less ettringite formation than OPC (Figure 8b). However, similar to BFS30_TW, BFS30_EW generated less ettringite than OPC, and it resulted in the smallest and most compact hydrate particle structure (Figure 8c) [41,42]. Similar characteristics were observed on day 10, although the amount of hydrates increased and their structure became more compact (Figure 9).

## 4. Conclusions

The strength and hydration characteristics of cement matrices produced using BFS and electrolyzed alkaline-reduced water were evaluated, and the following conclusions were drawn:

(1) The electrolyzed alkaline-reduced water of strong alkalinity of pH 12.5 to 13 led to a significant increase in mortar flexural strength and compressive strength compared to tap water. However, in the case of BFS50, the flexural strength of the mortar was not significantly affected despite the use of electrolyzed alkaline-reduced water.

BFS 30% exhibited a good overall compressive and flexural strength, and was 80 and 90 percent as strong as OPC on days 5 and 10, respectively. The effect of the electrolyzed alkaline-reduced water was similar to previous reports using a 5 M NaOH alkaline activator.

(2) X-ray diffraction analysis indicated that the Ca(OH)_2_ content of BFS 30% based on electrolyzed alkaline-reduced water was lower than OPC, although an almost identical cement matrix was produced. BFS 30% based on electrolyzed alkaline-reduced water exhibited a slightly higher Ca(OH)_2_ content than BFS 30% based on tap water.

(3) The scanning electron microscopy images revealed the formation of smaller and more compact hydrates in BFS 30% based on electrolyzed alkaline-reduced water compared to BFS 30% based on tap water. The amount of hydrates increased between days 5 and 10, and their structure became more compact.

This experimental study was limited to early aging (days 5 and 10), where the basic analytical approach evaluated the physical components and hydration characteristics of the cement matrices produced after electrolyzed alkaline-reduced water addition and BFS substitution. The use of electrolyzed alkaline-reduced water as binding water and 30% BFS replacement effectively improved the cement matrix properties. Further application of electrolyzed alkaline-reduced water to concrete and mortar is recommended.

## Figures and Tables

**Table 1:** Chemical compositions of the raw materials.

Materials	Chemical Composition (%)								L.O.I. ^1)^
	CaO	SiO_2_	Al_2_O_3_	Fe_2_O_3_	MgO	SO_3_	K_2_O	Others	
OPC ^2)^	60.3	19.8	4.9	3.3	3.8	2.9	1.1	0.9	3.0
BFS ^3)^	42.5	34.2	14.5	0.6	5.3	2.0	0.0	1.0	2.1

^1)^ L.O.I.: Loss on ignition.; ^2)^ OPC: Ordinary Portland cement; ^3)^ BFS: Blast furnace slag.

**Table 2:** Physical properties of the raw materials.

Material.	Properties
OPC	Type I Ordinary Portland Cement Density: 3150 kg/m^3^, Fineness: 330 m^2^/kg
BFS	Blast Furnace Slag Density: 2250 kg/m^3^, Fineness: 360 m^2^/kg
Fine Aggregate:	ISO Standard Sand, Particle Size: below 2 mm Fineness Modulus: 2.99, Density: 2620 kg/m^3^

**Table 3:** Experimental design.

Experimental Factors	Experimental Levels
Type of Binding Material	Ordinary Portland Cement, Blast Furnace Slag
Substitution Ratio of BFS (Binder × wt%)	30 and 50
Type of Binding Water	Tap Water and Electrolyzed Alkaline-Reduced Water
W/B	0.50
Curing Conditions	Temperature: 20 ± 2 °C Relative Humidity: 80 ± 5%
Test Items	Flexural Strength (MPa), Compressive Strength (MPa) SEM, XRD

**Table 4:** Mixing proportions of the mortar.

Sample	W/B	B:S ^1)^	Binder (g)		Water (g)	
			Cement	BFS	TW ^2)^	EW ^3)^
OPC	0.50	1:3	450		225	
BFS30_TW ^2)^	0.50	1:3	315	135	225	
BFS30_EW ^3)^	0.50	1:3	315	135		225
BFS50_TW	0.50	1:3	225	225	225	
BFS50_EW	0.50	1:3	225	225		225

^1)^ B:S = Binder (cement + BFS): Sand, Sand: ISO Standard sand; ^2)^ TW: Tap water; ^3)^ EW: electrolyzed alkaline-reduced water.

**Table 5:** Testing of the mortar hardening properties.

Evaluation Item	Test Method	Size (mm)
Flexural Strength (MPa)	ASTM 348 [36]	40 × 40 × 160
Compressive Strength (MPa)	ASTM C109/C109M [37]	40 × 40 × 160
Scanning Electron Microscope	ASTM C1723 [38]	
X-ray Diffraction	ASTM C1365 [39]	
